# Proteasome Activity and C-Reactive Protein Concentration in the Course of Inflammatory Reaction in Relation to the Type of Abdominal Operation and the Surgical Technique Used

**DOI:** 10.1155/2018/2469098

**Published:** 2018-10-14

**Authors:** Marzena Tylicka, Ewa Matuszczak, Maria Karpińska, Adam Hermanowicz, Wojciech Dębek, Halina Ostrowska

**Affiliations:** ^1^Department of Biophysics, Medical University of Białystok, Mickiewicza 2A, 15-089 Białystok, Poland; ^2^Department of Pediatric Surgery, Medical University of Białystok, Waszyngtona 17, 15-274 Białystok, Poland; ^3^Department of Biology, Medical University of Białystok, Mickiewicza 2A, 15-089 Białystok, Poland

## Abstract

Surgical tissue damage and the accompanying inflammatory response lead to proteasome activation, initiation of damaged protein degradation, and induction of acute-phase inflammatory response. The aim of this study was to investigate the rate of change in proteasome chymotrypsin-like (ChT-L) activity and C-reactive protein concentration depending on the degree of tissue damage and their correlation with prealbumin concentrations in children before and after abdominal surgery. This experimental study included children who underwent abdominal surgery between 2015 and 2017. Plasma prealbumin concentrations and C-reactive protein levels (CRP) were determined by standard biochemical laboratory procedures. Proteasome activity was assessed using a Suc-Leu-Leu-Val-Tyr-AMC peptide substrate. Elevation of plasma proteasome activity was noted in children after laparoscopic and open abdominal surgeries. However, 20S proteasome activity in children undergoing conventional open surgery was significantly higher (*P* < 0.05) than in patients subjected to laparoscopy. At the same time, an increase in the CRP level was observed. However, there was no correlation between C-reactive protein concentrations and the type of abdominal surgery while there was a correlation observed in the case of proteasomes. Proteasome activity correlates with the degree of surgical tissue damage and prealbumin concentrations. More invasive surgery leads to a stronger activation of the proteasome involved in removing proteins that were damaged due to the surgical procedure. Proteasomes are more specific markers because there is a correlation between proteasome activity and the type of abdominal surgery in contrast to C-reactive protein concentrations which are not different in response to surgery performed in regard to ovarian cysts or cholelithiasis.

## 1. Introduction

All types of trauma, including surgery, elicit a systemic response which is related to the severity of the injury [1]. Cytokines, especially tumor necrosis factor *α* (TNF-*α*), interleukin 1 *β* (IL-1*β*), and interleukin 6 (IL-6), are invoked within the immune system in response to surgical stress and infection [2, 3]. Moreover, IL-6 can regulate, in combination with TNF, the synthesis of acute-phase proteins such as the C-reactive protein (CRP) [4]. Numerous growth factors, cytokines, and chemokines are involved in the formation of new tissue and ultimately in wound closure after surgery. Furthermore, many studies also emphasize their role in the formation of surgical adhesions that arise from abnormal wound healing [5, 6].

There is growing evidence pointing to the role of the proteasome-dependent pathway in nuclear factor (NF-*κ*B) activation, modulation of cytokine levels, and thus in the process of wound healing [7]. Proteasomes activate NF-*κ*B by the degradation of its inhibitor and cause an increase in cytokine production in response to surgical stress [8]. The role of proteasomes in inflammatory response is designed to eradicate microorganisms, promote healing following injury, and restore homeostasis. Proteasomes can be responsible for an increase in protein catabolism during the acute phase response. Furthermore, they are also involved in proteolysis which aims to protect organisms against misfolded and damaged proteins arising from surgical tissue damage [9, 10].

The 20S proteasome is a major system for the removal of oxidized protein, while the 26S proteasome is deactivated under even mild degrees of oxidation. Oxidation damage to proteins can be caused by oxidants or reactive oxygen species produced during immune response. In this condition, 20S proteasomes are quite resistant. Therefore, they are important in the maintenance of normal cell function due to the proteolytic removal of damaged proteins accumulated within cells [11, 12].

All eukaryotic cells contain proteasomes. Recent studies have shown that proteasomes are identified not only in tissues but also in fluids, such as serum, cerebrospinal fluid, or extracellular bronchoalveolar lavage fluids. In physiological conditions, they are present in human blood plasma as circulating proteasomes and elevated in pathological conditions such as trauma or surgery. Taken together, regardless of location, they are responsible for maintaining homeostasis, as well as for coping with stress [12].

Laparoscopic surgery (LS) is replacing the open technique in many centers worldwide. LS is associated with less pain, more rapid recovery, a faster return to normal diet, and a reduction in hospitalization time. One of the other advantages of laparoscopy compared with open surgery is reduced inflammatory response [13].

The purpose of this novel human study was to characterize inflammatory response in children undergoing laparoscopic and open abdominal surgeries, by analyzing changes in selected inflammatory mediators: C-reactive protein concentrations, prealbumin levels, and circulating 20S proteasome activity following surgical tissue damage.

## 2. Materials and Methods

### 2.1. Patients

**Figure 1:**
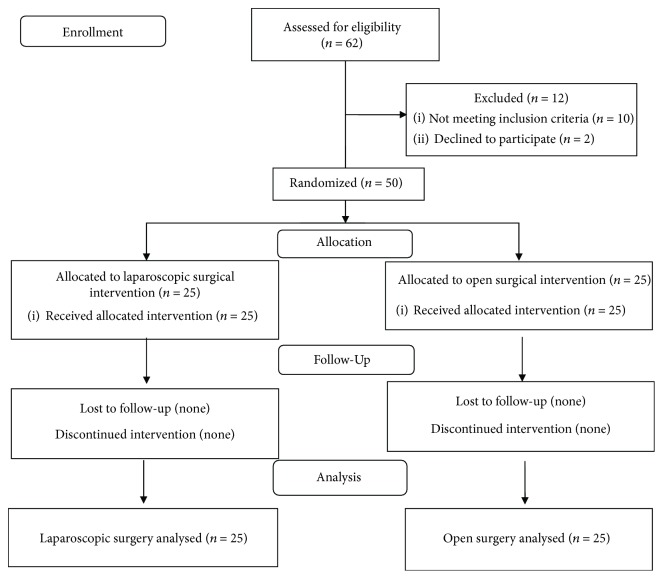
Flow diagram of patient enrollment.

Fifty from sixty-two children who were admitted to the Pediatric Surgery Department of the Medical University of Białystok for possible abdominal surgical procedures between 2015 and 2017 were eligible to participate in the study. Inclusion criteria were female or male patients diagnosed with cholelithiasis and ovarian cysts, aged between 7 and 17, who were directed to either laparoscopic or open surgery. Exclusion criteria were patients who had severe preexisting infections, immunological or cardiovascular diseases that required long-term medication, and existing intraoperative or postoperative complications. Those patients whose parents gave informed consent for both clinical and biochemical follow-up were randomly included in the study. Fifty patients were randomized and allocated to study groups with 25 in each group (Figure 1). Members of both groups were operated on due to cholelithiasis (*N* = 26) and ovarian cysts (*N* = 24).

Randomization was performed by way of a computer-generated schedule (randomization list created with the MS Excel random function), and the results were sealed in numbered envelopes which were opened after establishing that there were no exclusion criteria.

The most common clinical presentation in children referred for surgery was abdominal pain. Laparoscopic treatment of ovarian cysts was performed in patients with smaller cysts and for whom there was no risk that the change may be cancerous. Contraindication for this type of surgery was the incidence of postoperative adhesions after the removal of appendicitis in children.

For each patient, key data such as the type of surgical procedure, surgical duration, surgical findings, the presence of any different pathology, and demographic data including age and gender was collected. Patient demographics and baseline characteristics are summarized in Table 1 with *P* values which describe the comparability between the two study groups. There were no significant differences between variables (*P* > 0.05) except length of hospital stay, which differed significantly for patients undergoing laparoscopic and open surgeries (*P* < 0.05).

Patients after surgical intervention received standard surgical care and postoperative treatment according to the standard treatment protocols of our clinic; and after surgeries, they were given intravenous paracetamol which does not suppress the inflammation [14].

Approval for this study was obtained from the Local Ethics Committee.

### 2.2. Methods

Venous blood samples (1–2 mL) were drawn 4–8 h before and 6–8 h after surgical intervention with routine laboratory workup. Blood samples were collected in plasma test tubes containing EDTA as an anticoagulant, centrifuged at 1500*g* for 15 min at room temperature, and stored at −80°C before subsequent analysis.

Stored at low-temperature, samples before assay were thawed and centrifuged to remove fibrinogen.

The proteasome chymotrypsin-like activity in the plasma was assayed using the fluorogenic peptide substrate Suc-Leu-Leu-Val-Tyr-AMC in the presence of SDS (sodium dodecyl sulfate-selective enzyme activator), as described by Ma et al. and Tylicka et al. [15, 16]. The 20S proteasome cannot efficiently degrade peptides or proteins unless they are highly denatured; therefore, the 20S proteasome in plasma has been activated by the addition of sodium dodecyl sulfate. SDS function included facilitating infiltration of the protein substrate into the proteasome channel.

Plasma samples were activated for 15 min at room temperature with 5 *μ*L of 10% SDS. Sodium dodecyl sulfate concentration in the plasma activation phase was 1%. Subsequently, 10 *μ*L of samples was carried to reaction wells containing 30 *μ*L of an assay buffer (0.05% SDS in 100 mM Tris/HCl, pH = 7.5) and 10 *μ*L of the fluorogenic peptide AMC substrate, so that the total volume of the reaction mixture was 50 *μ*L and the concentration of SDS was 0.03% (the concentration needed for the maximal activation of the 20S proteasome). Suc-Leu-Leu-Val-Tyr-AMC is cleaved by the chymotryptic-like activity of the proteasome releasing free AMC (7-amino-4-methylcoumarin). Measurements of the quantity of the released AMC were carried out using a fluorescence microplate reader FLUOstar OPTIMA (BMG Labtech, Germany). The reaction mixture was incubated at 37°C in a fluorescence microplate reader, and the fluorescence was monitored after time intervals of 3 min each during a 60 min incubation period at an excitation wavelength of 355 nm and an emission wavelength of 460 nm. One unit of the 20S proteasome chymotrypsin-like activity was expressed as the amount of AMC released from the substrate per minute (pmol/min = U). Specific activity was expressed as a unit of the amount of total protein (U/mg) in which the concentration in plasma samples was determined by the Bradford method, using the Bio-Rad assay reagent with bovine serum albumin as the standard. All assays were performed in triplicate.

To confirm the specificity of the assay, the plasma was preincubated with the selective proteasome inhibitor epoxomicin (1.0 *μ*Mol/L) for 15 min before the addition of a substrate.

To confirm that addition of SDS caused a real increase in proteasome catalytic activity, we measured plasma 20S proteasome activity with the addition of sodium dodecyl sulfate and without.

CRP plasma level was measured by the latex-enhanced immunoturbidimetric method for the in vitro quantitative determination of CRP in human plasma on a Cobas Integra 800 analyzer (Roche Diagnostics, Penzberg, Germany). Measurement of prealbumin concentrations in the plasma of children operated on due to abdominal injury was carried out using an automated biochemistry analyzer, according to standard procedures.

### 2.3. Data Analysis

Statistical analysis was performed using the STATISTICA PL release 12.5 Program. All the results are presented as median with 25th and 75th percentiles. The results were analyzed by the Kruskal-Wallis and Mann–Whitney *U* test (two-sided nature) because the tested parameters in the plasma of patients did not pass the normality test. The data was previously tested for normality using the Shapiro-Wilk test. For multiple testing, Bonferroni correction was applied to compare differences between groups (three-fold pairwise testing; level of significance *P* < 0.017) (Table 2). Differences in baseline characteristics between the two study groups were tested using Chi-square and Fisher's exact test (categorical variables) or two-sample *t*-test (continuous variables) (Table 1). Correlations were studied by using the Spearman correlation test. Differences were considered significant where there was a value of *P* < 0.05 or *P* < 0.017 for the three-fold pairwise testing.

The primary outcome measure of this study was to compare postoperative inflammatory proteasome response among the groups after laparoscopic and open surgeries. The expected mean value of the proteasome activity in the two groups and the difference in outcome considered clinically important were estimated based on preliminary unpublished data of patients undergoing different types of surgery in our department in previous years (SD = 0.903; ES = 0.155). The estimated difference that was considered clinically significant was 20%. The sample size was calculated with an independent two-sided *t*-test by choosing a 95% confidence level, a margin of error of ±5%, a power of 80%, a difference of 20% between the 2 groups, the size of the pediatric population in our region, and the number of cases performed per year. Based on STATISTICA calculations, there should be 25 evaluable patients in each treatment group.

## 3. Results

**Figure 2:**
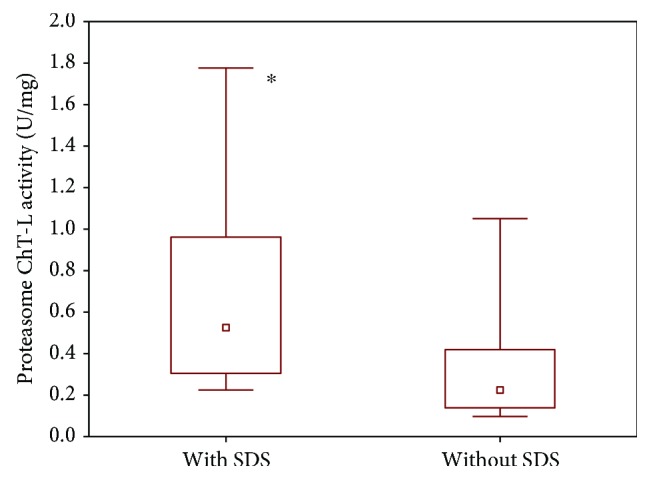
Plasma 20S proteasome chymotrypsin-like activity after SDS activation and without this agent in patients receiving open abdominal surgery (^∗^*P* = 0.023).

Comparison of the proteolytic activity of 20S after SDS activation and without the activation of this agent confirmed that the observed growth of the 20S activity is really an increase of proteasome catalytic activity. The 20S proteasome activity was statistically significantly higher in plasma after SDS activation than without using this activator (Figure 2).

An increase in the circulating proteasome activity was observed in patients after laparoscopic and open surgeries. The elevation of this parameter was statistically significant regardless of the type of surgical procedure; however, in the case of open surgery, the increase of 20S proteasome activity was twice as much. In patients undergoing laparoscopic abdominal surgery, proteasome chymotrypsin-like activity in plasma increased 101.22% relative to the value before the operation, while after open abdominal surgery, the increase relative to the value before surgical intervention was 193.08% (Table 2).

**Figure 3:**
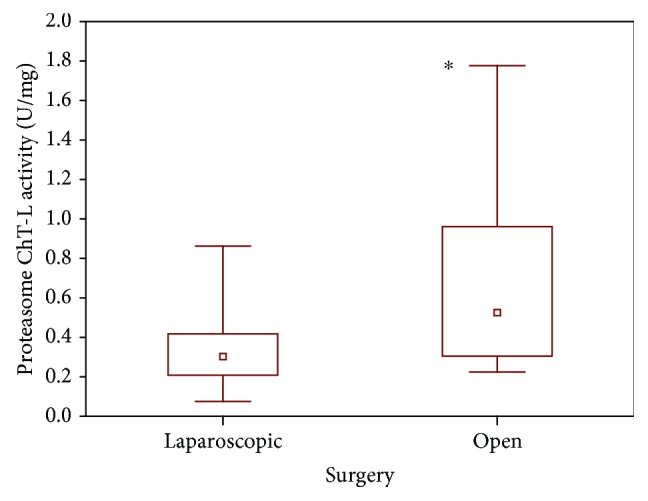
Plasma 20S proteasome chymotrypsin-like activity in patients after laparoscopic and open surgery (^∗^*P* < 0.017).

Compared to the laparoscopic techniques, the proteasome was more strongly activated during open abdominal surgery. There was a significant difference (*P* < 0.017) between circulating plasma proteasome activity in children undergoing laparoscopic (median, 0.30 U/mg; range, 0.21–0.42 U/mg) and open surgical procedures (median, 0.53 U/mg; range, 0.31–0.96 U/mg). Much higher proteasome activity was observed after the use of open surgery techniques (Figure 3).

**Figure 4:**
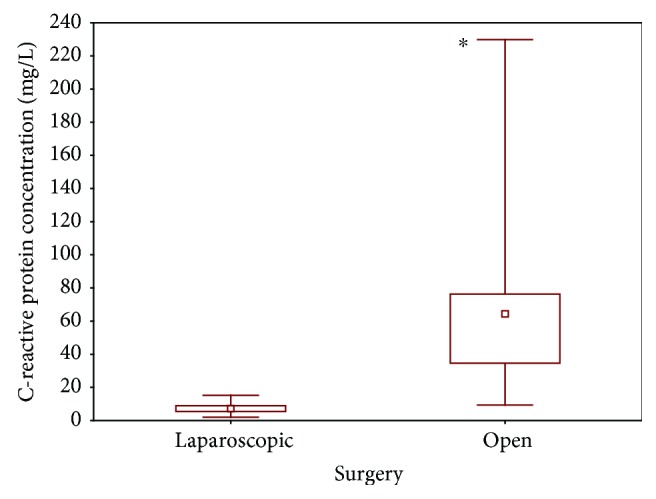
Plasma C-reactive protein concentration in patients after laparoscopic and open surgery (^∗^*P* < 0.05).

Surgical interventions caused an intensified acute-phase reaction characterized by an increase in the C-reactive protein level. We observed that, as in the case of proteasome activity, plasma CRP concentration is statistically significantly higher (*P* < 0.05) in children undergoing open abdominal surgeries (median, 64.35 mg/L; range, 34.6–76.3 mg/L) than in patients subjected to laparoscopic procedures (median, 7.05 mg/L; range, 5.4–9 mg/L) (Figure 4).

Assessing the impact of the type of abdominal surgery (cholelithiasis and ovarian cyst) or surgical procedure used (open surgery and laparoscopic surgery), we observed that the surgical technique has a stronger influence on C-reactive protein concentration (*r* = 0.815) than on proteasome activity (*r* = 0.385). The type of surgery has an influence only on proteasome activity (*r* = 0.458).

We found a statistically significant correlation between proteasome activity and prealbumin concentrations (*P* < 0.05), both before and after surgery. We found a stronger correlation between plasma 20S proteasome activity and prealbumin level after surgical intervention (*r* = −0.556 negative, high correlation) than before (*r* = −0.470 negative, average correlation). In contrast, we did not notice any significant correlations between C-reactive protein concentrations and prealbumin levels before and after surgery (*P* > 0.05).

## 4. Discussion

Surgical procedures, tissue dissection, and organ manipulation initiate an inflammatory response. Several authors have confirmed that this response is proportional to the tissue damage [17, 18]. A recent study by Majetschak et al. showed that the elevated activity of circulating 20S proteasomes reflects cellular tissue damage and might be a useful biomarker of disease severity and progression [19, 20]. Zoeger et al. postulated that 20S proteasomes are potential disease markers and their activation in patients suffering from various disorders such as systemic lupus erythematosus and rheumatoid arthritis may reflect the disease state and may form the basis for the development of a new diagnostic tool [21]. Our previous findings also suggested that plasma 20S proteasome activity could be an additional biomarker of tissue damage in the course of burn disease [22]. Moreover, proteasomes serve as an information-gathering mechanism for the immune system [23]. These statements are confirmed by Egerer et al. who observed that proteasomes represent novel sensitive markers of the autoimmune inflammatory processes and reflect the magnitude of cellular damage [24]. Their findings suggested that the postoperative increase of circulating proteasomes may support the hypothesis that the source of proteasomes is injured cells. Based on their study on the circulating 20S proteasome activity levels in patients with mixed connective tissue disease and systemic lupus erythematosus, Majetschak et al. pointed toward the release of 20S proteasomes from damaged tissue [20]. In contrast, when comparing trauma, sepsis, and abdominal surgery groups, Roth et al. demonstrated that increased 20S proteasome levels are caused by immunological activity rather than cellular damage [25].

The measurement of proteasomes in surgical patients undergoing open and laparoscopic surgeries is valuable due to the fact that proteasome inhibitors may be useful for modulating wound healing. Abnormal scarring is a source of significant morbidity, and thus, should constitute an active area of research. In the literature, there are reports which underline the utility of proteasome inhibitors in improving wound healing in a standard rat model. Currently available pharmaceutical therapy for scar prevention has no proven beneficial effects, has limited applications, or induces detrimental side effects [26]. Recent data has suggested that proteasome inhibitors in vitro profoundly alter human fibroblast metabolism, which favors the degradation of the overproduction of the extracellular matrix (ECM). Excessive deposition of ECM leads to altered tissue and organ architecture and in consequence to dysfunction and pathology. Several authors demonstrated that proteasome inhibitors have shown promise as potential antifibrotic agents in myelodysplasia, pulmonary fibrosis, cardiac fibrosis, multiple fibrotic models including renal fibrosis, and skin fibrosis [26]. Moreover, according to animal studies, the ubiquitin-proteasome system is involved in the formation of postsurgical peritoneal adhesions in rats. Bortezomib-treated rats showed a decreased number of peritoneal adhesions, decreased values of ubiquitin, and the 20S proteasome. Postoperative adhesions are a commonly occurring complication after surgery, which highlights the importance of conducting research on the proteasomes in surgical patients [6].

Our data demonstrated that C-reactive protein concentration also increased in the case of laparoscopic and open surgeries. Similar results were obtained by Halevy et al. [27]. Laparoscopy is less traumatic than conventional open surgery because of a minor incision, less tissue disruption, minimal manipulation, and decreased postoperative pain [28]. In our study, we also observed that C-reactive protein concentration was significantly higher in children undergoing open abdominal surgery than a laparoscopic procedure (Figure 4).

There is some evidence that the 20S proteasome complex is responsible for the recognition and degradation of oxidized proteins [29]. A recent study by Arsalani-Zadeh showed that a higher degree of oxidative stress occurs after open surgery [30]. Aging, oxidative stress, and other pathological conditions are accompanied by the accumulation of oxidatively modified proteins. Our observation is similar to results obtained by Bukan et al. who suggested that laparoscopy causes less oxidative stress than open surgery [31]. In the current study, we noted statistically significant differences in plasma 20S proteasome activity between children undergoing laparoscopy and open abdominal surgery (Figure 3). We found that plasma 20S proteasome chymotrypsin-like (ChT-L) activity is higher after open surgery than after laparoscopic intervention. A decreased level of oxidized proteins may have an impact on the lower ChT-L proteasome activity in the plasma of children undergoing laparoscopic abdominal procedures.

Comparing both markers, the proteasome and C-reactive protein, we found that the type of surgery has an influence on the circulating 20S proteasome activity in contrast to C-reactive protein concentration which was not different in response to surgery due to an ovarian cyst or cholelithiasis. Our results support the concept presented by Allin and Nordestgaard that CRP is an unspecific marker of inflammation, and individuals with slightly elevated CRP levels have an increased risk of several diseases and of all-cause mortality [32]. Several lines of evidence strongly suggested that the acute-phase response is not diagnostic for any particular disease, but occurs as a response to several pathological conditions and diseases [33]. Our earlier findings showed that the proteasome may be a biomarker of tissue damage—more severe in the group of burn patients in comparison to patients with mild head injury and blunt abdominal trauma [34, 35]. Also, the last study showed that correlation between proteasome activity and length of surgery in contrast to the C-reactive protein indicated that CRP is only an indicator of the pathological state, while the function of the proteasome is more complex due to its participation in the oxidative stress-induced repair of tissue damage [16].

Based on these findings, we examined the correlation between proteasome activity and prealbumin concentrations before and after surgery to prove that proteasomes are involved in protein degradation. A study of prealbumin levels after gastric surgery by Bae et al. also showed that early postoperative prealbumin levels decreased by approximately 50% of preoperative values [36]. We found an average negative (*r* = −0.470) correlation between proteasome ChT-L activity and prealbumin concentrations in childrens' plasma before surgery and a strong negative correlation (*r* = −0.556) after surgery. We observed that the negative correlation between proteasome activity and prealbumin levels is stronger after than before surgery, which confirms the fact that surgical intervention induces proteasome function. In contrast, we did not find any statistically significant correlation between C-reactive protein concentrations and prealbumin levels before and after surgery (*P* > 0.05). These observations may indicate that CRP, as opposed to the proteasome, is not involved in the protein degradation process.

## 5. Conclusion

Surgical trauma affects not only C-reactive protein concentration, commonly used in laboratory practice, but also proteasome activity.

Plasma proteasome activity in the context of the surgical technique used reflects the degree of tissue damage, and in comparison to C-reactive protein (CRP) may be a specific biomarker of the oxidative stress and inflammation. Proteasomes are strongly activated by injury exerted by conventional open surgery compared to laparoscopy due to the greater tissue damage. In contrast to CRP, the type of surgery has an influence on 20S proteasome activity. The C-reactive protein is a nonspecific inflammatory mediator which is significantly increased postoperatively, regardless of the type of operation.

Monitoring of the proteasome activity can help in further diagnostic and therapeutic decisions and more effective treatment. The control of the proteasome function by using their inhibitors may lead to faster recovery by improving repair and wound healing processes and preventing the formation of postsurgical adhesions which are a common complication after surgery. Bearing in mind the above observations, we strongly believe that the future may very likely lead to the clinical application of the results of studies on proteasomes and its correlation with surgically treated inflammatory processes located in the abdominal cavity.

## Figures and Tables

**Table 1:** Characteristics of the study patients.

	Enrollment and randomization (*N* = 50)		*P* value^∗^
The type of surgical procedure	Laparoscopic *N* = 25	Open *N* = 25	

Gender (F/M)	Male	*N* = 14 (48.28%)	*P* = 0.529
	Female	*N* = 36 (51.72%)	
	Male *N* = 8 (32%)	Male *N* = 6 (24%)	
	Female *N* = 17 (68%)	Female *N* = 19 (76%)	

Age	7–17 y (median, 13) (range, 11–15)		*P* = 0.521
	8–17 y (median, 12)	7–17 y (median, 14)	

Type of surgery	Ovarian cyst	*N* = 24 (48%)	*P* = 0.388
	Cholelithiasis	*N* = 26 (52%)	
	Ovarian cyst *N* = 11 (44%)	Ovarian cyst *N* = 13 (52%)	
	Cholelithiasis *N* = 14 (56%)	Cholelithiasis *N* = 12 (48%)	

Operation times	1.5 h–2.5 h		*P* = 0.078
	1.5 h *N* = 15 (60%)	1.5 h *N* = 9 (36%)	
	2.5 h *N* = 10 (40%)	2.5 h *N* = 16 (64%)	

Type of anesthesia	General anesthesia *N* = 50 (100%)		

Blood loss (mL)	Nonsignificant	Nonsignificant	

Intraabdominal pressure	12 mmHg	N/A	

Intraoperative/postoperative complications	None	None	

Type and dosage of medication	All patients after surgical intervention received standard surgical care and postoperative treatment according to the standard treatment protocols of our clinic and after surgeries were given intravenous paracetamol^∗∗^		

Length of stay	3–4 days		*P* = 0.036
	Up to 3 days *N* = 12 (68.42%)	Up to 3 days *N* = 5 (26.09%)	
	Up to 4 days *N* = 13 (31.58%)	Up to 4 days *N* = 20 (73.91%)	

^∗^A *P* value < 0.05 is considered to show a significant difference between groups. ^∗∗^Paracetamol has no influence on inflammatory response

**Table 2:** The statistical parameters of proteasome 20S ChT-L activity in the plasma of children before and after abdominal surgery performed using laparoscopic and open technique.

Proteasome 20S ChT-L activity (U/mg)	Laparoscopic surgery (*N* = 25; 50%)		Open surgery (*N* = 25; 50%)	
	Before	After	Before	After
Median	0.15	0.30	0.18	0.53
Minimum	0.05	0.08	0.01	0.23
Maximum	0.56	0.86	0.38	1.78
Percentiles (25%–75%)	0.10–0.23	0.21–0.42	0.14–0.33	0.31–0.96
*P* value^∗^	*P* = 0.009		*P* = 0.001	
Increase (%)^∗∗^	**101.22**		**193.08**	
*P* value^∗^	**P** = 0.003			

^∗^A *P* value < 0.017 is considered to show a significant difference between groups (according to Bonferroni correction). ^∗∗^The increase in proteasome activity after surgery was calculated relative to the value before the operation.

## Data Availability

The data used to support the findings of this study are available from the corresponding author upon request.
